# Knee acoustic emissions as a noninvasive biomarker of articular health in patients with juvenile idiopathic arthritis: a clinical validation in an extended study population

**DOI:** 10.1186/s12969-023-00842-7

**Published:** 2023-06-20

**Authors:** Quentin Goossens, Miguel Locsin, Sevda Gharehbaghi, Priya Brito, Emily Moise, Lori A Ponder, Omer T Inan, Sampath Prahalad

**Affiliations:** 1 School of Electrical and Computer Engineering, Georgia Institute of Technology, Technology Square Research Building, 85 Fifth St NW, Atlanta, GA 30308 USA; 2 Department of Pediatrics, Emory University School of Medicine, Atlanta, GA 30223 USA; 3 Children’s Healthcare of Atlanta, Atlanta, GA 30223 USA; 4 Department of Human Genetics, Emory University School of Medicine, Atlanta, GA 30223 USA

**Keywords:** Juvenile idiopathic arthritis, Joint acoustic emissions, Supervised machine learning, Knee Joint Health, Digital Biomarker

## Abstract

**Background:**

Joint acoustic emissions from knees have been evaluated as a convenient, non-invasive digital biomarker of inflammatory knee involvement in a small cohort of children with Juvenile Idiopathic Arthritis (JIA). The objective of the present study was to validate this in a larger cohort.

**Findings:**

A total of 116 subjects (86 JIA and 30 healthy controls) participated in this study. Of the 86 subjects with JIA, 43 subjects had active knee involvement at the time of study. Joint acoustic emissions were bilaterally recorded, and corresponding signal features were used to train a machine learning algorithm (XGBoost) to classify JIA and healthy knees. All active JIA knees and 80% of the controls were used as training data set, while the remaining knees were used as testing data set. Leave-one-leg-out cross-validation was used for validation on the training data set. Validation on the training and testing set of the classifier resulted in an accuracy of 81.1% and 87.7% respectively. Sensitivity / specificity for the training and testing validation was 88.6% / 72.3% and 88.1% / 83.3%, respectively. The area under the curve of the receiver operating characteristic curve was 0.81 for the developed classifier. The distributions of the joint scores of the active and inactive knees were significantly different.

**Conclusion:**

Joint acoustic emissions can serve as an inexpensive and easy-to-use digital biomarker to distinguish JIA from healthy controls. Utilizing serial joint acoustic emission recordings can potentially help monitor disease activity in JIA affected joints to enable timely changes in therapy.

## Background

Juvenile idiopathic arthritis (JIA), a heterogenous group of childhood arthritides, is the most common childhood rheumatologic condition [1]. JIA causes significant morbidity across the globe and affects many joints and extra-articular organs [2].

While diagnosing JIA early in its disease process is crucial for disease modification and treatment [3–5], multiple diagnostic challenges limit ideal management, including significant shortages of pediatric rheumatologists in the US [6]. Magnetic resonance imaging (MRI), while more sensitive and specific than the clinical exam, is costly and time consuming, limiting its application [7]. Furthermore, no easy-to-use and inexpensive objective measurement exists for monitoring joints affected by JIA in the long term, apart from symptom-based questionnaires that can be affected by subjectivity [8].

A potential step in ameliorating these challenges is the development of new diagnostic modalities to define, diagnose, and longitudinally monitor articular involvement in JIA. Here, joint acoustic emissions (JAEs) have the potential to change this status quo. JAEs are the sounds that originate from the movement of the articulating surfaces of the joints, and therefore, vary based on the articulatory properties of the joint. JAEs are affected by joint diseases such as rheumatoid arthritis [9–11] or osteoarthritis [12], which change the tribological properties of the joint [13]. When novel machine learning algorithms are leveraged to analyze these sounds, JAEs can be used as digital acoustic biomarkers that are easy to obtain using non-invasive, inexpensive, and compact equipment applied to the surface of the skin. This information can be exceptionally valuable to inform pediatric rheumatologists toward efficient personalized treatment. Our group previously demonstrated that JAEs have merit in assessing knee joint health in adult populations [9, 14, 15]. We have also demonstrated the feasibility of JAEs to discriminate JIA from healthy controls [10, 11]. Although these proof-of-concept studies demonstrated the potential of using JAEs as a tool to identify and monitor JIA, they were conducted in a relatively small study population (n < 45).

The knees are the most involved joint in JIA [16–18]; in a study of 95 newly diagnosed children with JIA, knee involvement was seen in 74% at onset and 93% after 5 years [18]. The present study builds on our previous work on the use of JAEs as a biomarker to identify knee involvement in JIA. To validate our previous results, we recorded knee JAEs from a larger cohort compared to our previous studies. We also investigated the use of new machine learning classifiers. Specifically, we used JAEs to assess and differentiate knee health in children with active versus inactive JIA involvement. Here, we report on a more generalizable model for knee health assessment in children with JIA that offers clinical perspective compared to our previous work.

## Findings

### Methods

#### Subjects and data collection

JAEs from knees were recorded in Children’s Healthcare of Atlanta’s Center for Advanced Pediatrics. Children diagnosed with JIA were included in the study, while those with prior musculoskeletal injury were excluded. Each knee was analyzed separately from its pair. Knees were considered “active” if the knee had swelling alone, and/or tenderness and limited range of motion, at the time of recording on physical exam by an experienced attending rheumatologist. “Inactive” knees did not meet the active involvement criteria at the time of the recording.

**Fig. 1:**
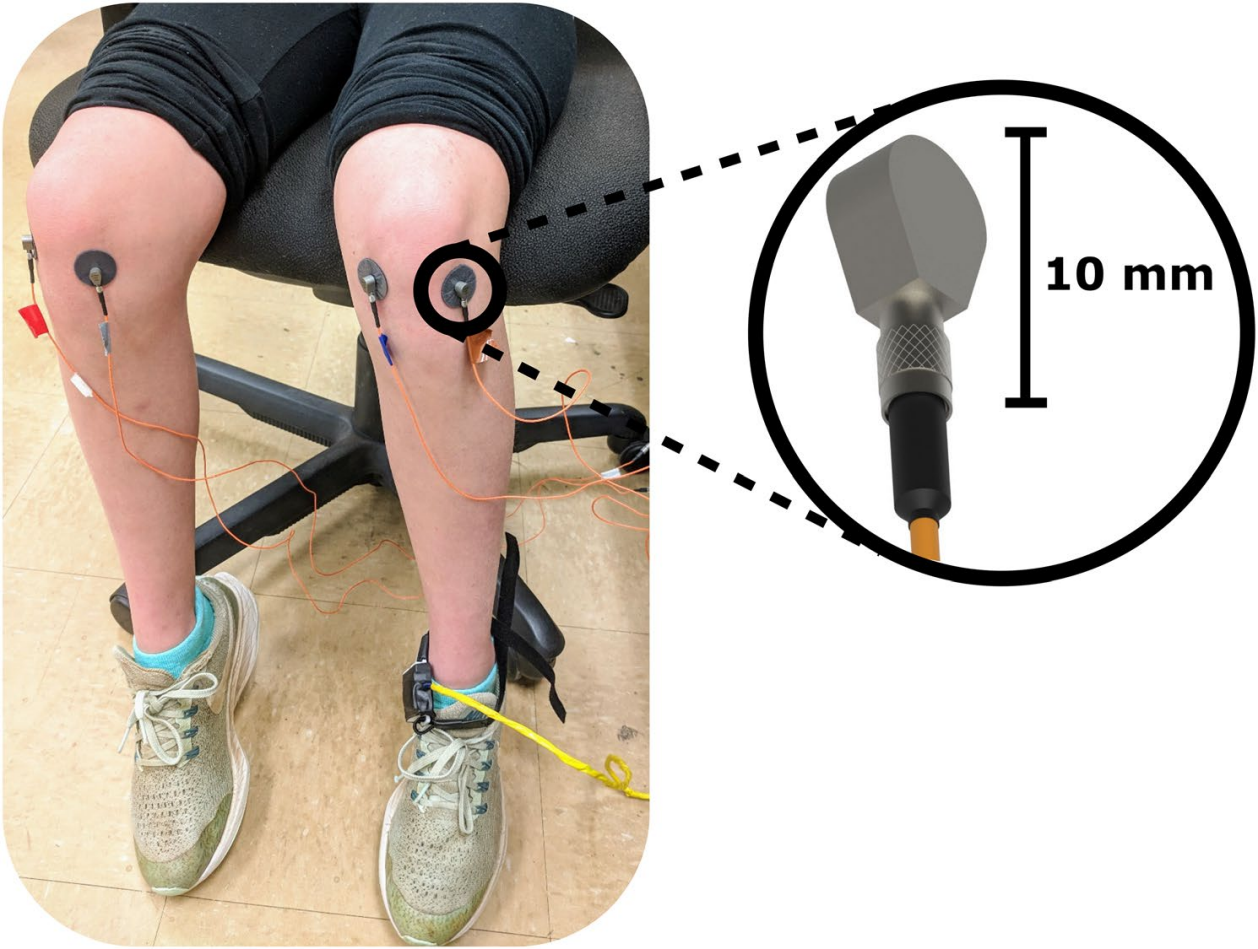
Overview of the experimental setup that was used to record JAEs. Two uniaxial accelerometers were used to record the joint sounds from the knee. A data acquisition system tethered to a laptop (not shown in figure) was used to sample and save the analog acoustic emission signals

All subjects performed ten seated, unloaded flexion-extension cycles, one cycle every four seconds guided by an instructional cartoon as previously described (10, 11). JAEs were recorded from both knees using two miniature uniaxial accelerometers (3225F7, Dytran Instruments Inc., CA, USA) attached 2 cm medial and lateral to the distal patellar tendon using double-sided tape (Rycote Microphone Windshields Ltd, Stroud, Gloucestershire, UK). These accelerometers have a broad bandwidth (2 Hz – 10 kHz), high sensitivity (100 mV/g), and low noise floor (0.0007 grms). A data acquisition system (USB-4432, National Instruments, TX, USA) sampled the joint sounds at 100 kHz. Figure 1 shows an overview of the described experimental setup to record JAEs.

#### Signal processing & machine learning model

The recorded JAE signals were divided into flexion-extension cycles. Wavelet denoising and bandpass filtering (100 Hz − 12 kHz) were used to remove noise from the signals. After denoising, we divided the JAE cycles into segments of 200 ms with 50% overlap. 273 time-frequency audio features were extracted from each segment and the average and standard deviation of each feature were calculated from all segments of the 10 flexion-extension cycles. A principal component analysis was performed on these 273 features to extract the 60 top principal components that described 95% of the variance of the data. These 60 principal components were used as input features for an Extreme Gradient Boosting (XGBoost) machine learning classifier. Knees that were labeled as active JIA were considered to have a stronger label than inactive JIA knees and were therefore used to train the classifier. This set of active JIA data was supplemented with 80% of the healthy controls to be our training data set. We tested our trained classifier on the remaining data; all inactive knees and the remaining 20% of the healthy controls. The classifier predicted a joint score between 0 and 1, which can be considered as the probability of having JIA. Leave-one-leg-out cross-validation (LOLO-CV) was used to assess the performance of the classifier on the training set. Joint scores, overall accuracy, confusion matrices, sensitivity / specificity, receiver operating characteristic (ROC) curve and corresponding area under the curve (AUC) are reported. All signal preprocessing was performed using Matlab (MathWorks, MA, USA) and the machine learning classifier was developed in Python using the scikit-learn toolbox. A two-sample Kolmogorov-Smirnov test was used to compare the distributions of the joint scores of the active and inactive JIA knees.

## Results

**Fig. 2:**
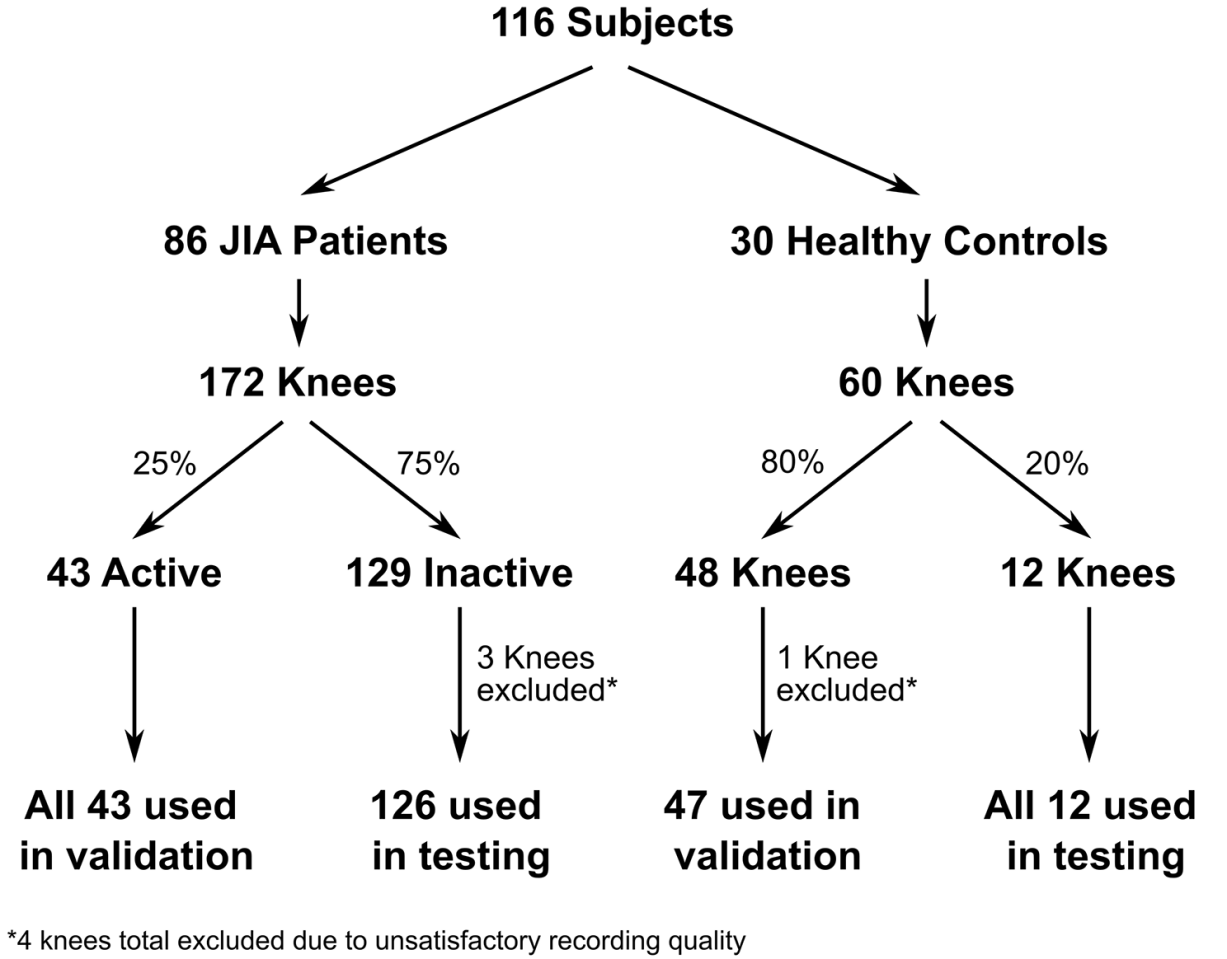
Schematic overview of the study population of the presented work

A total of 116 children participated in this study, (86 with JIA diagnosed by a pediatric rheumatologist and 30 age and gender matched controls, Fig. 2**and** Table 1). Of the 172 knees of patients with JIA, 43 knees were considered active, and 129 knees were inactive recordings. The 30 healthy controls were examined by a pediatric rheumatologist and had no evidence of arthritis.

**Table 1:** Overview of the subjects that were recruited for the presented study

	*n*	Age [years] mean (min - max)	Male	Female	Duration of Disease [years] median (1st, 3rd quartiles)
Total Subjects	116	12.5 *(5–20)*	32 (27.6%)	84 *(72.4%)*	N/A
JIA Subjects	86 *(74.1%)*	12.7 *(5–20)*	22 (25.6%)	64 *(74.4%)*	3.41 (1.91, *7.44)*
Subtype					
*Oligoarthritic*	39 *(45.3%)*	11.4 *(5–19)*	10 *(25.6%)*	29 *(74.4%)*	4.17 (2.16, *7.77)*
*Polyarthritis RF Negative*	18 *(21.0%)*	12.6 *(6–20)*	1 *(5.6%)*	17 *(94.4%)*	3.68 (2.24, *6.33)*
*Polyarthritis RF Positive*	5 *(5.8%)*	15.2 *(11–19)*	0 *(0.0%)*	5 *(100.0%)*	1.73 (1.44, *1.85)*
*Enthesitis Related Arthritis*	10 *(11.6%)*	13.0 *(8–20)*	6 *(60.0%)*	4 *(40.0%)*	3.81 (1.92, *5.16)*
*Psoriatic*	3 *(3.5%)*	17.0 *(16–18)*	0 *(0.0%)*	3 *(100.0%)*	*14.9 (9.58, 15.1)*
*Systemic*	6 *(7.0%)*	13.5 *(7–18)*	2 *(33.3%)*	4 *(66.7%)*	4.78 (3.21, *6.64)*
*Undifferentiated*	5 *(5.8%)*	15.4 *(10–18)*	3 *(60.0%)*	2 *(40.0%)*	2.47 (0.24, *3.36)*
Controls	30 *(25.9%)*	12.1 *(7–18)*	10 (33.3%)	20 *(66.7%)*	N/A

**Fig. 3:**
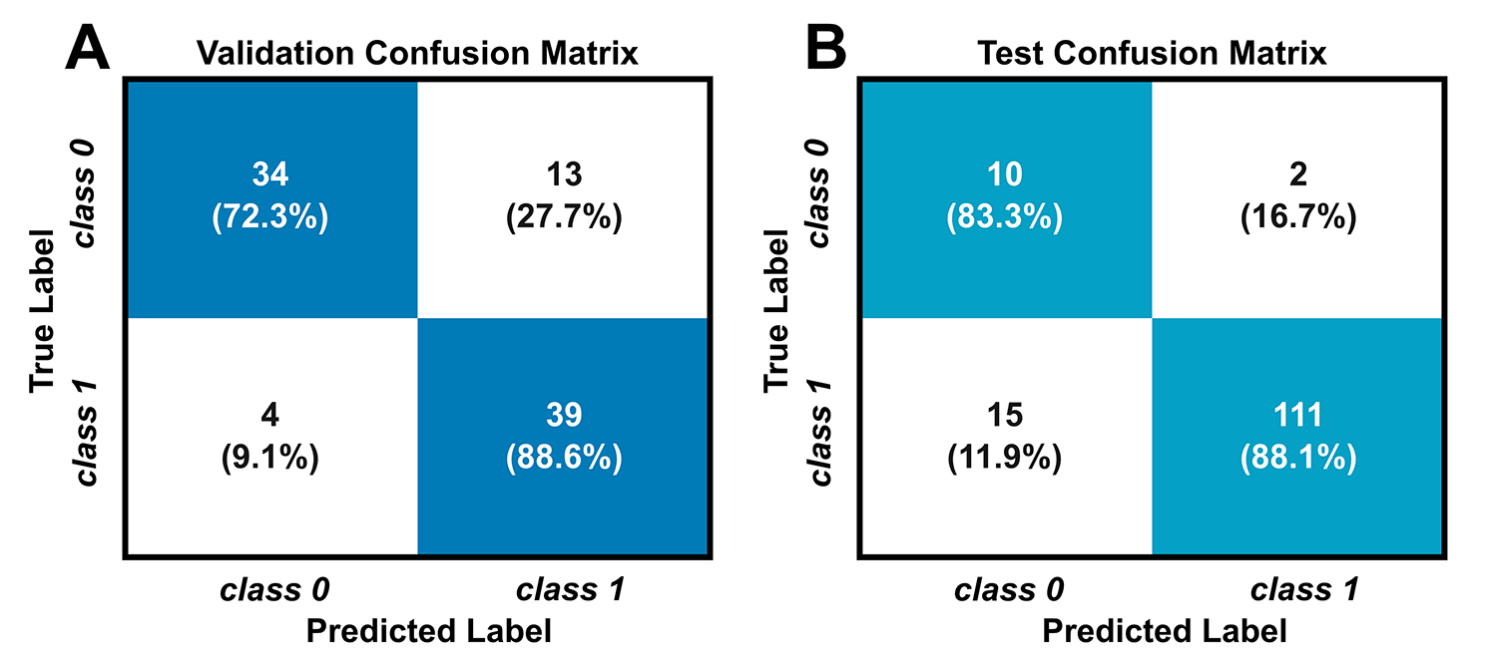
(**A**) The confusion matrix of the cross-validation on the training data set and (**B**) the confusion matrix corresponding to the test data set

**Fig. 4:**
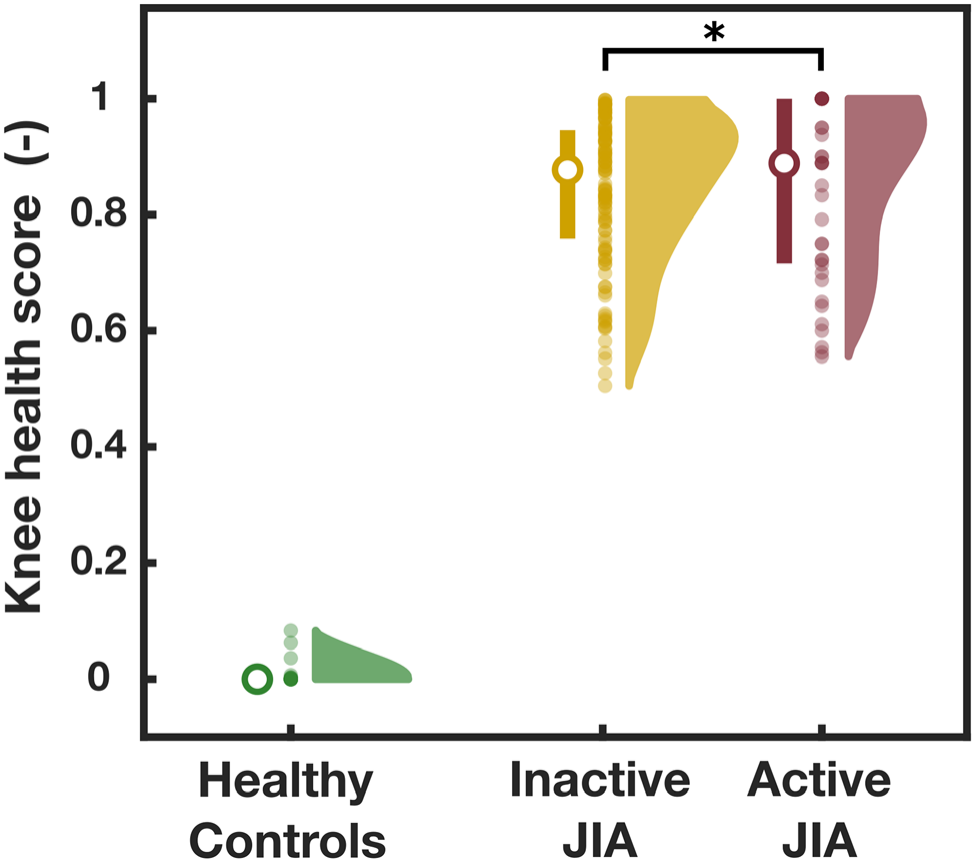
Density distributions of the joint scores from healthy, inactive, and active JIA knees. From left to right for each group: median and 1st / 3rd quartiles, scatter plot of all joint scores, and corresponding joint score distributions

**Fig. 5:**
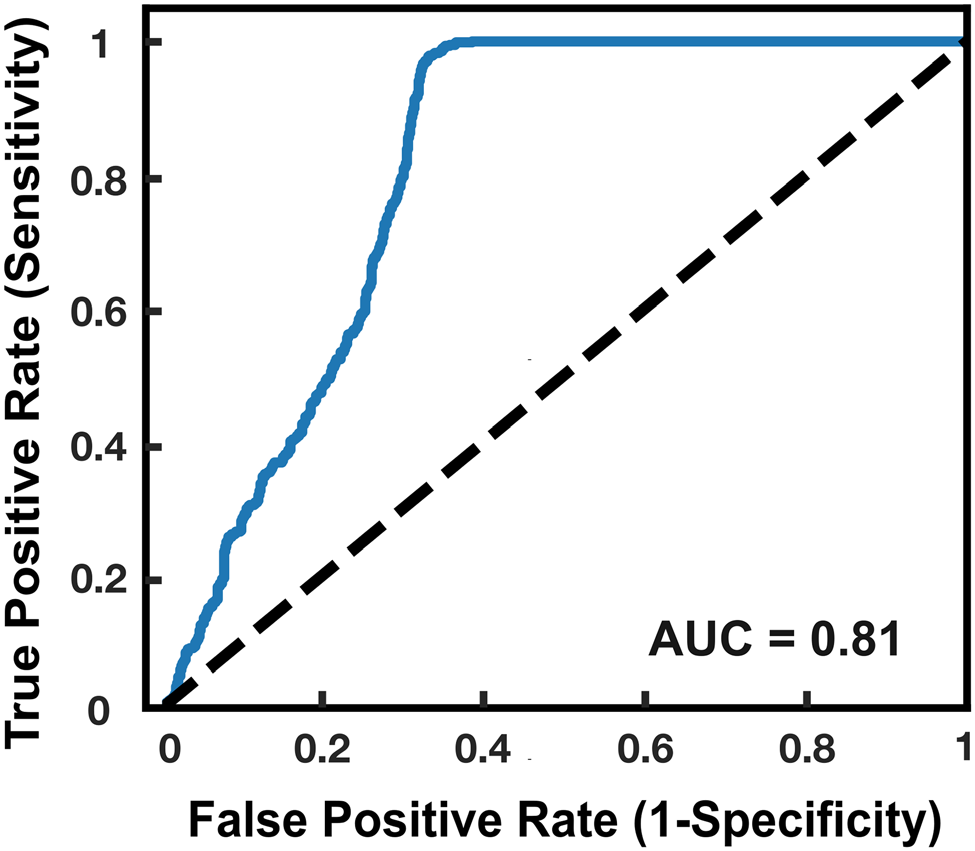
Receiver Operating Characteristic (ROC) curve and corresponding area under the curve (AUC) of the classifier obtained for the cross-validation

The LOLO-CV on the training set of the classifier resulted in an 81.1% accuracy, while the classifier resulted in an 87.7% accuracy for the testing data set (inactive JIA + healthy controls). The LOLO-CV and testing confusion matrices are shown in Fig. 3. The sensitivity and specificity of the LOLO-CV on the training data set were 88.6% and 72.3%, respectively. The sensitivity and specificity of the testing data set were 88.1% and 83.3%, respectively. Figure 4 shows the distribution of the joint scores of the correctly labeled healthy controls, active and inactive JIA knees separately. The distributions of the joint scores of the active and inactive knees were significantly different (p < 0.05). The ROC curve and corresponding AUC for the cross-validation is shown in Fig. 5.

## Discussion & conclusions

We present a study on a larger cohort of 116 children building further on our previous work on knee JAEs as a non-invasive and convenient tool to detect JIA [10, 11]. We introduced the use of a new machine learning classifier, Extreme Gradient Boosting (XGBoost), which generalized well on an extensive test data set. Moreover, this new classifier led to a better separation between children with JIA and healthy controls compared to the classifier (logistic regression) we used in our previous work. This is reflected by the joint scores of the healthy control knees which were consistently close to 0 (Fig. 4), representing the high sensitivity of the recorded JAEs.

The differences in joint acoustic emissions (JAEs) between inactive and healthy knees can be attributed to chronic inflammation and scarring in inactive knees, resulting in thickened synovium and altered JAEs. This is not observed in healthy knees, especially in children, who do not have these features associated with articular inflammation in JIA.

The presented technology utilizes JAEs as a digital biomarker for assessing joint health, which clinicians can easily implement it in outpatient settings for screening and monitoring diseases. This tool can aid general pediatricians in making referrals to rheumatologists, prompt early treatment [3–5], and facilitate decisions on diagnostic tests and treatment adjustments. Additionally, wearable devices incorporating this digital biomarker may serve as future tools for at-home disease monitoring.

Limitations of this study include the inherent subjectivity of active and inactive labeling by the physical exam of providers, potentially decreasing the accuracy of validation of the machine learning classifier. No significant difference was found between the median joint scores of active and inactive knees, even though their distributions were different. The subjects tested in our study also varied in their courses of treatment, potentially masking the accuracy of the classification labels made by patients and their doctors. More objective labeling by a validated gold standard method (e.g., radiologic diagnostics, including MRI, ultrasound, or inflammatory markers in the laboratory), and longitudinal monitoring could improve the classification algorithm and reveal significant differences between active and inactive joint scores. Future directions of JAE studies should also include disease and joint score correlation in other joints affected by JIA.

The presented work demonstrates the feasibility to use JAEs as a non-invasive digital biomarker for articular health assessment in JIA. The use of JAEs by clinicians in the outpatient setting, therefore, represents a potentially inexpensive and easy-to-use screening or disease monitoring tool to help decrease and quantify disease morbidity caused by JIA.

## Data Availability

The raw data supporting the conclusions of this article will be made available by the authors, without undue reservation.

## List of Abbreviations

AUC: Area Under the Curve
JAE: Joint Acoustic Emission
JIA: Juvenile Idiopathic Arthritis
LOLO-CV: Leave-One-Leg-Out Cross-Validation
MRI: Magnetic Resonance Imaging
ROC: Receiver Operating Characteristic
RF: Rheumatoid Factor

## References

[1] Zaripova LN, Midgley A, Christmas SE, Beresford MW, Baildam EM, Oldershaw RA. Juvenile idiopathic arthritis: from aetiopathogenesis to therapeutic approaches. Pediatr Rheumatol. 2021 Dec;19(1):135. 10.1186/s12969-021-00629-8 PMC8383464 34425842

[2] Petty RE, Laxer RM. Juvenile Idiopathic Arthritis: Classification and Basic Concepts.

[3] Nigrovic PA, Review. Is there a window of opportunity for treatment of systemic juvenile idiopathic arthritis? Arthritis & Rheumatology. 2014 Jun;66(6):1405–13. 10.1002/art.38615 24623686

[4] Tynjala P, Vahasalo P, Tarkiainen M, Kroger L, Aalto K, Malin M et al. Aggressive Combination Drug Therapy in Very Early Polyarticular Juvenile Idiopathic Arthritis (ACUTE-JIA): a multicentre randomised open-label clinical trial. Annals of the Rheumatic Diseases. 2011 Sep 1;70(9):1605–12. 10.1136/ard.2010.143347 21623000

[5] Wallace CA, Ringold S, Bohnsack J, Spalding SJ, Brunner HI, Milojevic D, et al. Extension study of participants from the trial of early aggressive therapy in Juvenile Idiopathic Arthritis. J Rheumatol. 2014 Dec;41(12):2459–65. 10.3899/jrheum.140347 25179849

[6] Correll CK, Spector LG, Zhang L, Binstadt BA, Vehe RK. Barriers and alternatives to pediatric rheumatology referrals: survey of general pediatricians in the United States. Pediatr Rheumatol. 2015 Dec;13(1):32. 10.1186/s12969-015-0028-6 PMC4517495 26215389

[7] Malattia C, Damasio MB, Magnaguagno F, Pistorio A, Valle M, Martinoli C et al. Magnetic resonance imaging, ultrasonography, and conventional radiography in the assessment of bone erosions in juvenile idiopathic arthritis. Arthritis Rheum 2008 Dec 15;59(12):1764–72. 10.1002/art.24313 19035414

[8] Consolaro A, Ruperto N, Bazso A, Pistorio A, Magni-Manzoni S, Filocamo G et al. Development and validation of a composite disease activity score for juvenile idiopathic arthritis. Arthritis Rheum 2009 May 15;61(5):658–66. 10.1002/art.24516 19405003

[9] Richardson KL, Teague CN, Mabrouk S, Nevius BN, Ozmen GC, Graham RS, et al. Quantifying Rheumatoid Arthritis Disease Activity using a Multimodal sensing knee Brace. IEEE Trans Biomed Eng. 2022 Dec;69(12):3772–83. 10.1109/TBME.2022.3177074 35604995

[10] Whittingslow DC, Zia J, Gharehbaghi S, Gergely T, Ponder LA, Prahalad S, et al. Knee acoustic emissions as a Digital Biomarker of Disease Status in Juvenile Idiopathic Arthritis. Front Digit Health. 2020 Nov;19:2:571839. 10.3389/fdgth.2020.571839 PMC8521909 34713044

[11] Gharehbaghi S, Whittingslow D, Ponder LA, Prahalad S, Inan O. Acoustic emissions from loaded and unloaded knees to assess Joint Health in patients with juvenile idiopathic arthritis. IEEE J Biomed Health Inform. 2021;1–1. 10.1109/JBHI.2021.3081429 34003759

[12] Ewart D, Draisey A, Inan OT, Nichols C, Ozmen GC. Acoustic Classification of Early and Advanced Osteoarthritis of the Knee in Clinical Settings. ACR Convergence 2022; 2022 Nov 14; Philadelphia, PA, USA.

[13] Gharehbaghi S, Jeong HK, Safaei M, Inan OT. A feasibility study on Tribological Origins of knee acoustic emissions. IEEE Trans Biomed Eng. 2022 May;69(5):1685–95. 10.1109/TBME.2021.3127030 PMC9132215 34757899

[14] Ozmen GC, Safaei M, Semiz B, Whittingslow DC, Hunnicutt JL, Prahalad S, et al. Detection of Meniscal tear Effects on Tibial Vibration using Passive knee sound measurements. IEEE Trans Biomed Eng. 2021 Jul;68(7):2241–50. 10.1109/TBME.2020.3048930 PMC8284919 33400643

[15] Richardson KL, Gharehbaghi S, Ozmen GC, Safaei MM, Inan OT. Quantifying Signal Quality for Joint Acoustic Emissions using graph-based spectral embedding. IEEE Sens J 2021 Jun 15;21(12):13676–84. 10.1109/jsen.2021.3071664 PMC8516116 34658673

[16] Crayne CB, Beukelman T. Juvenile idiopathic arthritis. Pediatric clinics of North America. 2018 Aug;65(4):657–74. 10.1016/j.pcl.2018.03.005 30031492

[17] Eng SWM, Aeschlimann FA, van Veenendaal M, Berard RA, Rosenberg AM, Morris Q, et al. Patterns of joint involvement in juvenile idiopathic arthritis and prediction of disease course: a prospective study with multilayer non-negative matrix factorization. Sheikh A, editor. PLoS Med. 2019 Feb;26(2):e1002750. 10.1371/journal.pmed.1002750 PMC6390994 30807586

[18] Hemke R, Nusman CM, van der Heijde DMFM, Doria AS, Kuijpers TW, Maas M, et al. Frequency of joint involvement in juvenile idiopathic arthritis during a 5-year follow-up of newly diagnosed patients: implications for MR imaging as outcome measure. Rheumatol Int. 2015 Feb;35(2):351–7. 10.1007/s00296-014-3108-x 25119829

